# DEMENTIA SEVERITY AND QUALITY OF LIFE OF CARE PARTNERS OF OLDER ADULTS WITH DIABETES AND DEMENTIA

**DOI:** 10.1093/geroni/igae098.3072

**Published:** 2024-12-31

**Authors:** Oluwaseun Adeyemi, Caroline Blaum, Thaddeus Tarpey, Victoria Dickson, Joshua Chodosh

**Affiliations:** New York University Grossman School of Medicine, New York, New York, United States; NCQA, Ann Arbor, Michigan, United States; New York University Grossman School of Medicine, New York, New York, United States; University of Connecticut, Storrs, Connecticut, United States; New York University Grossman School of Medicine, New York, New York, United States

## Abstract

Caring for persons living with dementia (PLWD) is known to adversely affect care partner health. Health impacts specific to care partners of PLWD who also have diabetes is unknown. To examine this issue further, we assessed the association between dementia severity and care partner quality of life when caring for PLWD with diabetes. We extracted cross-sectional care partner survey responses enrolled in the Enhanced Quality in Primary Care for Elders with Diabetes and Dementia study (N=311). We defined dementia severity as a three-level categorical variable (mild, moderate, severe) using the Dementia Severity Rating Scale. Quality-of-life outcome measures were treatment burden score (scored 0 to 15; higher score = higher burden) and physical and mental health state (scored 0 to 20; higher score = better health). We controlled for both care partner and PLWD’s relationship, age, sex, race/ethnicity, and educational level. We performed multivariable quantile regression analyses and reported adjusted median difference (aMD) in quality-of-life measures (plus 95% confidence interval (CI)). Mean (standard deviation) ages of PLWD and their care partners were 81.5 (7.4) and 55.2 (12.4) years, respectively. Care partners were predominantly women (77.5%) and non-Hispanic Whites (38.9%). Compared to care partners of those with mild dementia, care partners of persons with severe dementia had more treatment burden (aMD: 1.41; 95% CI: 0.49, 2.32), and poorer mental health (aMD: -2.34; 95% CI: -3.60, -1.08). Caring for PLWD with diabetes exerts substantial burden on care partners especially when dementia is more severe and may benefit from more diabetes-focused education and support.

